# Five-Year Trends in Emergency Medicine Match Results and Future Outlook

**DOI:** 10.5811/westjem.47915

**Published:** 2025-09-27

**Authors:** Alexander Y. Sheng, Erin L. Simon, Timothy Friedmann, Eddie Garcia, Vytas Karalius, Michael Kiemeney, Brian Merritt, Brian Milman, Meghan Mitchell, Jared Mugfor, Mihir Patel, Rachel Wong, Esther H. Chen

**Affiliations:** * Warren Alpert Medical School at Brown University, Department of Emergency Medicine, Providence, Rhode Island; † Northeast Ohio Medical University, Cleveland Clinic Akron General Emergency Medicine Program, Akron, Ohio; ‡ Icahn School of Medicine at Mount Sinai, Department of Emergency Medicine, New York, New York; § Stanford University School of Medicine, Department of Emergency Medicine, Palo Alto, California; ¶ University of Texas Southwestern Medical Center, Department of Emergency Medicine, Dallas, Texas; || Loma Linda University School of Medicine, Department of Emergency Medicine, Loma Linda, California; # University of Utah, Department of Emergency Medicine, Salt Lake City, Utah; ** Northwestern University Feinberg School of Medicine, Department of Emergency Medicine, Chicago, Illinois; †† Drexel University College of Medicine, Department of Emergency Medicine, Philadelphia, Pennsylvania; ‡‡ Icahn School of Medicine at Mount Sinai, Mount Sinai Morningside West, New York, New York; §§ University of California, San Francisco, Department of Emergency Medicine, San Francisco, California

## BACKGROUND

Emergency medicine (EM) as a specialty attracts medical students due to its acuity of care, diversity of patients, pathologies, and procedures, accompanied by a uniquely flexible work-life balance. Except for a slight drop in 2014, the number of applicants to EM had steadily increased yearly since 2001, peaking in 2021 with 3,734 applicants.^1^ In fact, prior to 2022 EM was so competitive that it had fewer than 30 unmatched residency spots per year for over a decade.^2^

## THE BOTTOM FALLS OUT

**Figure 1:**
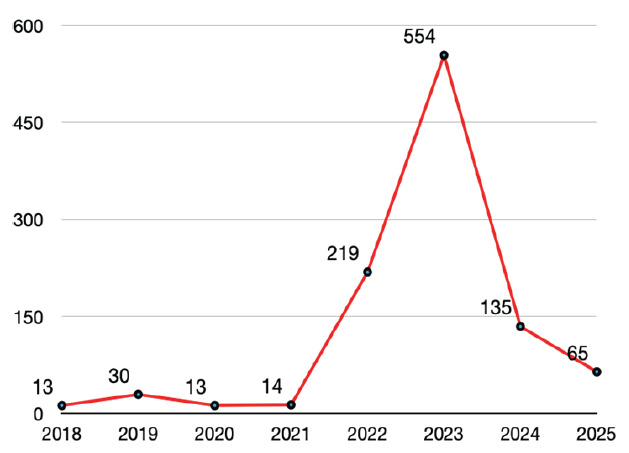
Unfilled emergency medicine Match positions, 2018–2025.

In 2022, the National Resident Matching Program (NRMP) EM Match resulted in a drastic fall in the number of applicants by 17.5% from 3,734 in 2021 to 3,081 in 2022, leading to 219 unfilled positions. This trend culminated in 2023, when the number of applicants dropped by an additional 10.3% to 2,765. Before the Supplemental Offer and Acceptance Program, 554 (18%) of 3,010 available positions went unmatched, leaving 132 (46%) of EM programs unfilled (Figure 1).^1^ Emergency medicine suddenly faced an unprecedented number of unfilled residency positions for the first time in decades, sparking concerns about the future of the EM workforce.^2^

This recent sharp decline in medical student interest in EM was likely influenced by multiple factors. In 2021, a workforce study by the American College of Emergency Physicians (ACEP) projected that by 2030 there could be almost 8,000 more emergency physicians than would be needed in the United States.^3^ The authors cited multiple reasons for predicting an workforce oversupply, including expansion of medical school class sizes, rapid growth in the number of EM residencies, lower population growth rate, and increasing participation by advanced practice clinicians (APC) in the workforce.^3^ This projection shocked the EM community, raising alarm about the future job market for emergency physicians. In 2023, a joint statement by ACEP suggested multiple reasons behind the decreasing number of EM applicants, including COVID-19, corporatization of medicine, emergency department (ED) boarding, economic challenges, and workforce projections.^4^

Also contributing to the increase in unfilled residency positions was the drastic rise in the number of residency programs, which could not be attributed solely to the transition from three accrediting organizations (the American Osteopathic Association [AOA], American Association of Colleges of Osteopathic Medicine, and Accreditation Council of Graduate Medical Education [ACGME]) to a single accreditation system within the ACGME. From 2001–2023, the number of EM residency programs increased from 117 to 287,^1^,^5^ with an overall increase in residency positions from 1,001 to 3,010.^1^ Although the increase in EM programs and positions was mirrored by the increase in applicants for two decades, this trend became unsustainable with the sudden drop in applicants from 2021 to 2023.

## THE REBOUND

Emergency medicine is a resilient specialty. Following the 2023 Match cycle, educators and program leadership at the local and national levels went to work to gather data, modify recruitment and interview strategies, and identify ways to prevent another match like 2023. An *EM Match Taskforce* was established with representatives from major EM organizations to evaluate and address the factors that led to the suboptimal Match results in 2023.^4^ Data suggested that several factors—having unfilled positions in the 2022 Match; smaller program size; Mid-Atlantic or East North Central location; corporate ownership; and prior AOA and more recent accreditation (within the prior five years)—were all associated with unfilled spots in 2023.^6^,^7^ Based on this information, programs were deliberate in the way that they recruited, interviewed, and ranked applicants to maximize their chance of filling their residency positions.^8^

Concurrently, the ACEP EM Workforce Taskforce worked to prioritize patient care and education over business interests, increase support for emergency care in rural communities, ensure adequate supervision for advanced practice clinicians, and expand the scope of EM practice to provide acute unscheduled care outside the traditional walls of the ED.^9^ Nationwide efforts to address ED boarding, reduce profit-driven practices in staffing of APCs, resist the growth of private equity in medicine, and mitigate burnout are ongoing.^10^,^11^

**Figure 2:**
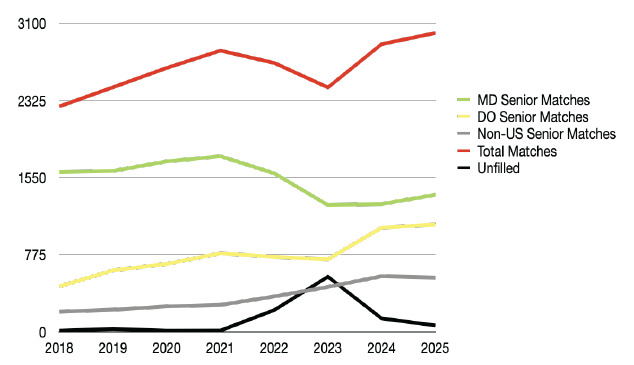
Five-year emergency medicine Match composition trends 2018–2025. *MD*, doctor of medicine; *DO*, doctor of osteopathic medicine; *US*, United States

As a result of these multipronged efforts, EM as a specialty has seen a rebound since 2023 (Figure 2). Going into the 2024 match, there was cautious optimism. The number of EM applications rose by 29.3% from 2023, totaling 3,574 applicants for 3,026 spots in 292 programs. While the number of positions continued to rise (2,665 in 2020, 2,840 in 2021, 2,921 in 2022, and 3,010 in 2023), the rate of expansion has slowed with 16 additional positions in 2024. The rise in applications was mainly driven by the increases in applications from osteopathic medical school students (DO) (by 43.9%) and international medical graduates (IMG) (by 81.6%). In contrast, there was a 15.4% decrease in the number of senior applicants from allopathic programs (MD) from 2020 to 2024, although there was a slight increase of 3.7% between 2023 and 2024.^1^

The 2024 Match results improved accordingly from 2023. Of the 3,026 available positions, 2,891 (95.5%) were filled with only 135 open positions in 2024 as compared to 554 in 2023. There were 1,386 MD senior applicants (38.8% of total applicants); of those, 1,285 matched into EM, which accounted for 44.4% of the filled spots. This compared to 2023 in which 1,337 MD seniors applied and 1,274 of 2,456 (51.9%) spots were filled by MD seniors.^1^ A total of 1,047 of 1,171 DO seniors who applied to EM successfully matched, representing 36.2% of the filled spots. In 2023, DO seniors accounted for 29.7% of positions filled.^1^

In 2024, there were 486 US IMG and 349 non-US IMG applications, totaling 835 IMG applicants. This represented a significant increase from 2023, with 487 total IMG applicants (366 US IMG, 121 non-US IMG). Thus, IMGs filled 448 positions (325 US IMGs and 123 non-US IMGs) in 2024, which accounted for 15.5% of the filled spots.^2^ In 2023, there were 350 positions filled by IMGs (14.3% of positions filled). In 2023, 71.9% of IMG applicants matched, but in 2024 this fell to 53.7%. (Table 1).

## THE 2025 MATCH

The 2025 EM Match marked another year-over-year improvement. There were 3,753 applicants for 3,068 positions from 292 programs, an increase from the 3,574 applicants for 3,026 positions in 2024. The vast majority (3,003, 97.9%) of the positions were filled, leaving 65 open spots—much fewer than the 554 and 135 unmatched positions in the 2023 and 2024 Match.^1^ In 2025, 1,514 US MD seniors applied to EM, an increase of 128 students compared to 2024. Allopathic seniors accounted for 1,377 (45.9%) of the total filled spots, a marginal increase from 1,285 (44.4%) in 2024. However, this is still far below the peak of 1,765 (62.5%) in 2021.^1^

Applicants by seniors from US osteopathic medical schools accounted for 1,231 of 3,753 (32.8%) EM applicants, and 1,078 of them matched into EM, representing 35.9% of the total filled positions. This is stable from the 2024 season, in which 1,047 US osteopathic school seniors accounted for 36.2% of the total positions filled; but it is much higher than the 2021 Match, during which only 790 US osteopathic seniors matched into EM, representing 28% of the total filled positions.^1^

**Figure 3:**
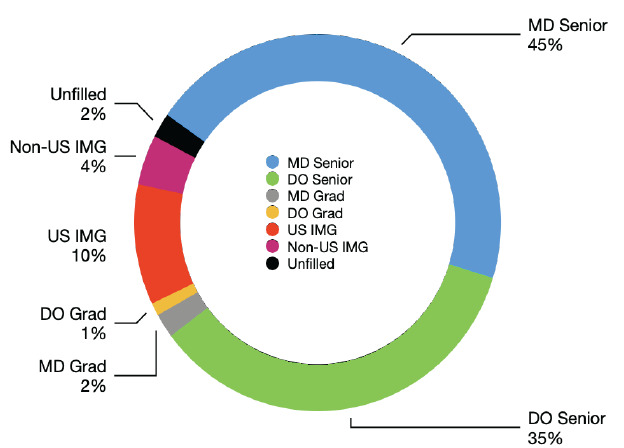
2025 emergency medicine Match composition. *MD*, doctor of medicine; *DO*, doctor of osteopathic medicine; *US*, United States; *IMG*, international medical graduate

A total of 467 US IMGs and 336 non-US IMGs applied to EM in the 2025 NRMP Match, with 315 and 131 matching successfully, respectively. The US and non-US IMGs together filled 446 positions (14.6%), essentially unchanged from the 448 positions they filled in 2024 (14.8%)^1^ (Figure 3).

## FUTURE IMPLICATIONS

The 2025 NRMP EM Match demonstrated a continued reduction in the number of unfilled positions, since the peak in 2023. While the positions offered were still primarily filled by seniors from US allopathic medical schools (46%), this proportion has largely remained steady in the last three years and has yet to return to 2021 levels (62%). Of the remaining 1,628 EM positions, US DO seniors filled 66%, with the last third occupied by US IMG and non-US IMG seniors. These proportions have remained consistent over the last two Match cycles. It is too early to tell whether EM has reached a new steady state of EM-bound medical school graduates.

A recent development that could impact Match results in the near future is the ACGME proposed program requirements for EM intended to prepare trainees for successful practice for the next 25 years.^12^ If the proposed change in program length occurs, which would require all programs to provide 48 months of training, it could transiently dampen interest in EM as a specialty for some students, specifically those who come from lower socioeconomic status or prioritize lifestyle and income. While this may make EM less competitive in the short term, the numbers will likely rebound in future years with a potential shift in the type of applicants who apply, as students with greater commitment to EM will likely still choose the specialty.

Studies of US allopathic school seniors who chose or considered EM as a specialty cited various factors that played a role in their ultimate choice. These included lifestyle factors,^13^ concerns regarding the unpredictability of shiftwork, lack of flexibility in practice setting and scope, mistreatment and violence against ED staff, a potentially diminishing job market for graduates, burnout and career longevity, EM’s standing in the healthcare landscape,^14^ and negative experiences of women clinicians who were perceived as not fitting the “EM stereotype.”^15^ A common thread to these studies is that a student’s EM clerkship experience significantly impacts their ultimate decision to pursue EM. Therefore, emergency clinicians and educators have a unique opportunity to leverage their interactions with medical students on their EM clerkship to share experiences and perspectives on these factors, while highlighting the positive, mission-driven, and service-oriented aspects of our specialty.

Emergency medicine continues to see an increase in the number of positions offered compared to the growth in the number of applications, resulting in unfilled positions. Confidence in the job market did improve in 2022, with 92% of physicians reporting a high likelihood of finding a job.^16^ However, concerns about a potentially diminishing job market in the future for emergency physicians persist from the physician workforce report published four years ago,^3^ with only 50% of physicians reporting a high likelihood of finding a desirable job and 44% reporting high confidence in a future job.^16^ In light of continued uncertainty, the EM workforce may benefit from limiting the expansion of class sizes and the creation of new programs over the short term, except in non-metro areas where there are consistent estimates of physician shortages.^17^

## CONCLUSION

The number of unfilled positions continued to decrease in 2025 from the peak in 2023. The positions filled by US MD seniors remain mostly stagnant as compared to 2021 levels. Leaders in emergency medicine remain cautiously optimistic after a successful 2025 Match. However, to maintain the interest of medical students, EM must maintain momentum to keep the job market sustainable and clinical practice fulfilling for current and future physicians.

## Figures and Tables

**Table 1:** Results from the emergency medicine Match 2020–2025.

Year	Programs	Positions offered	Total applicants	Total matches	Unfilled programs	Open spots	Percentage filled
2020	256	2,665	3,323	2,652	7	13	99.5
2021	273	2,840	3,734	2,826	9	14	99.5
2022	277	2,921	3,081	2,702	69	219	92.5
2023	287	3,010	2,765	2,456	132	554	81.6
2024	292	3,026	3,574	2,891	52	135	95.5
2025	292	3,068	3,753	3,003	27	65	97.9
